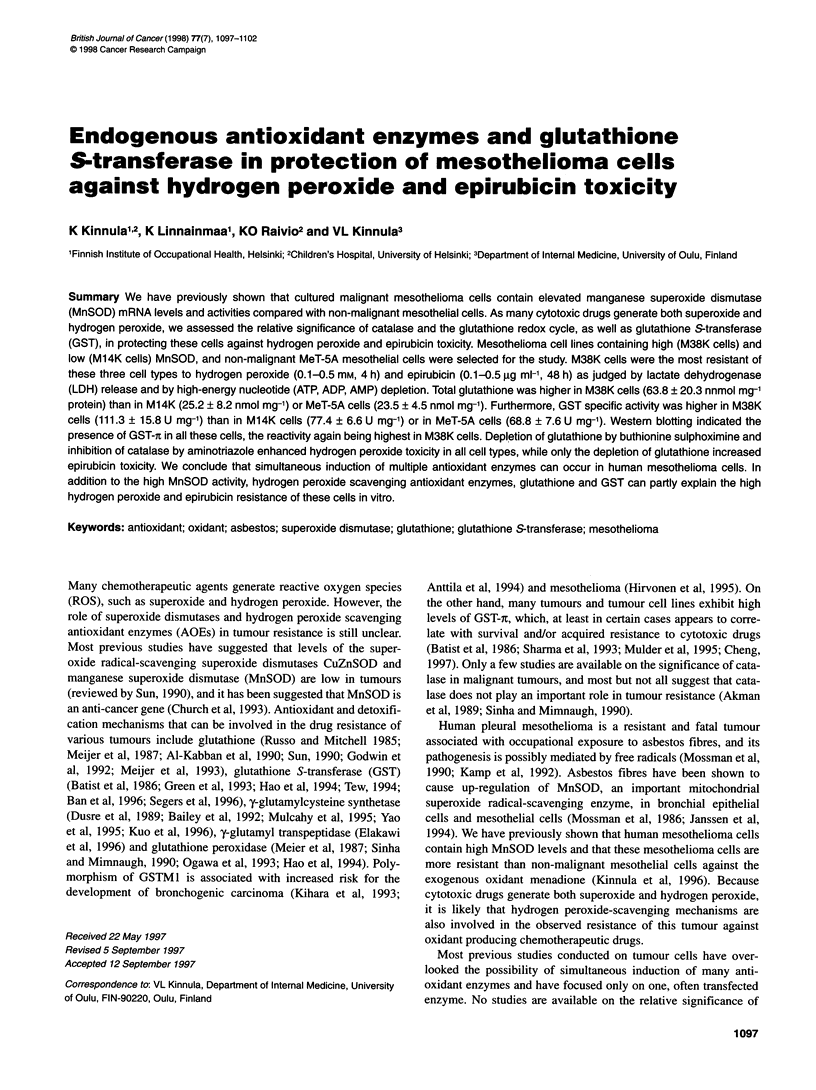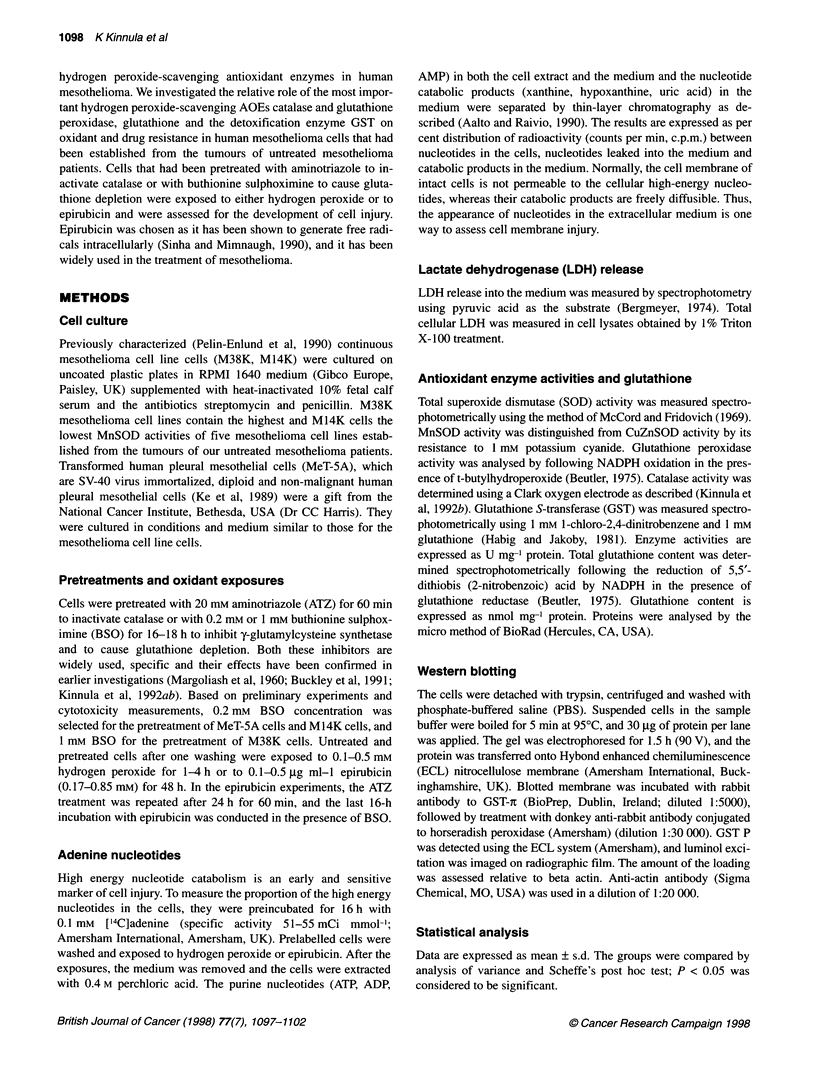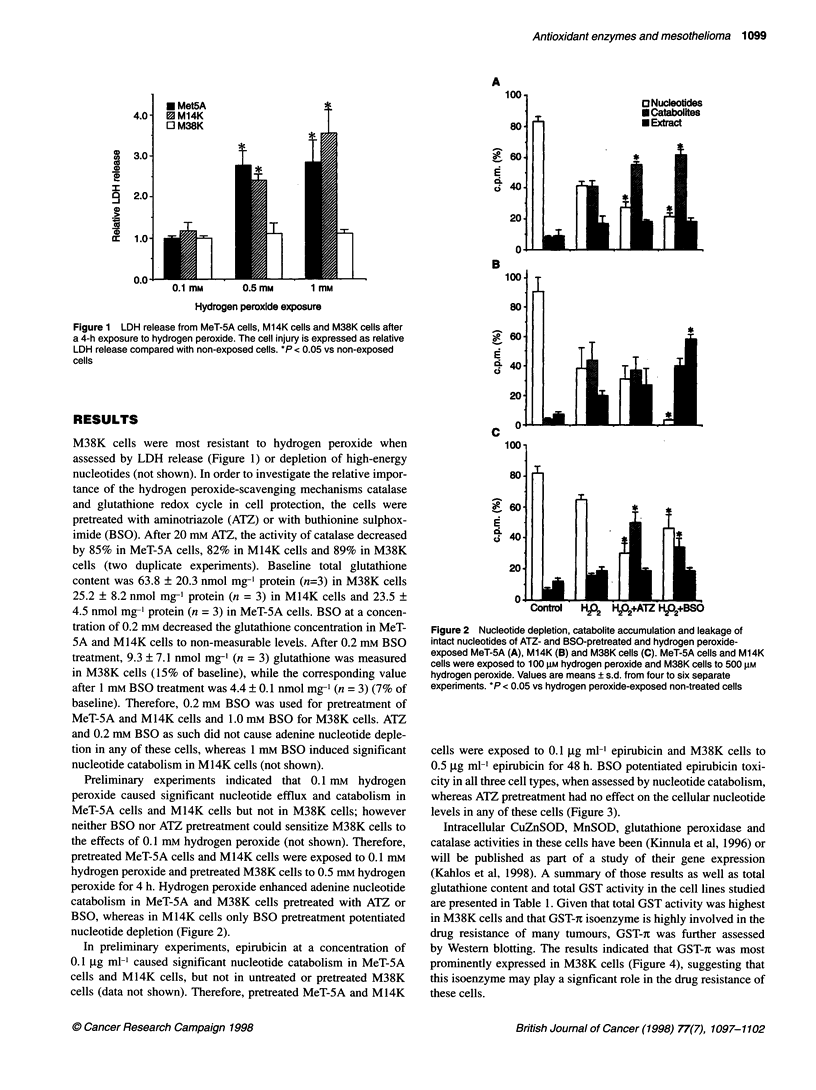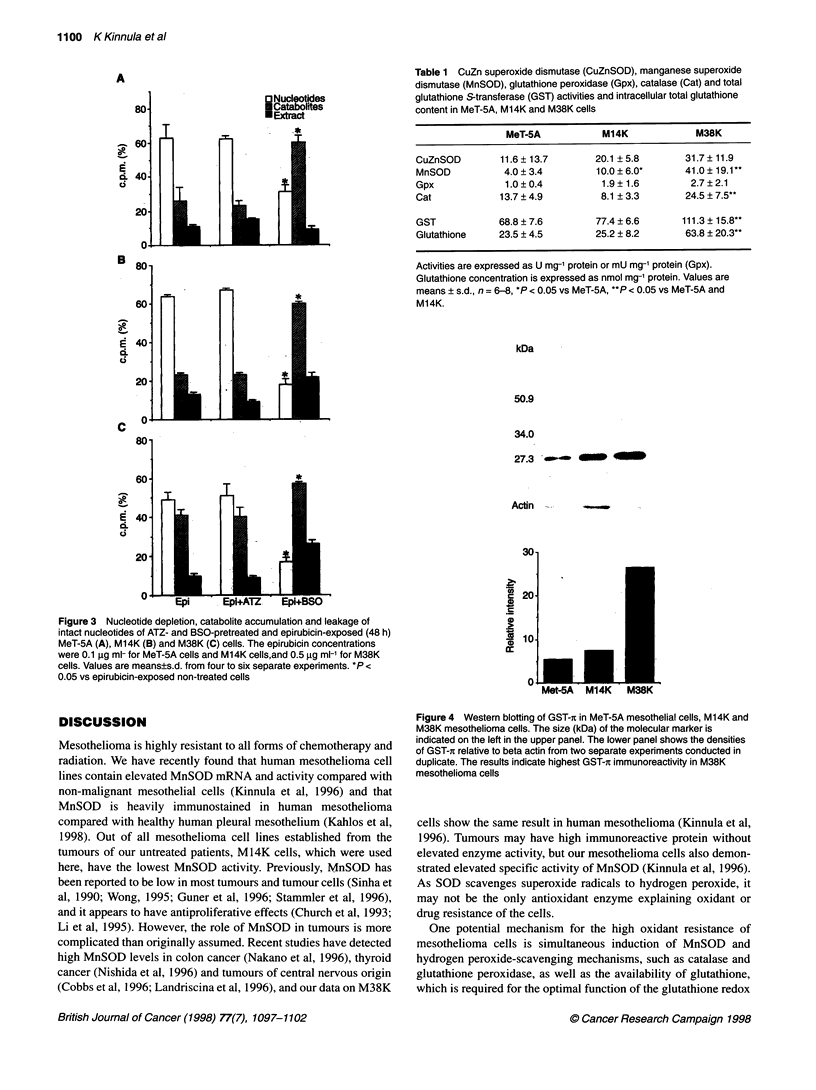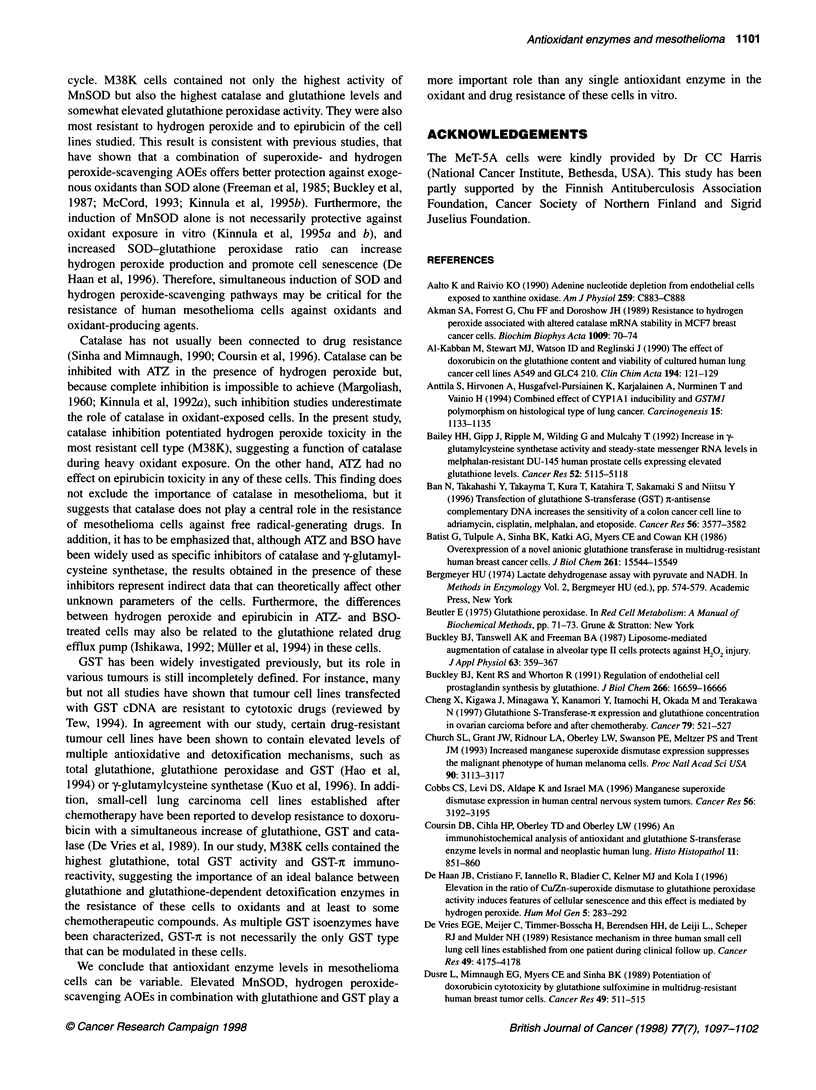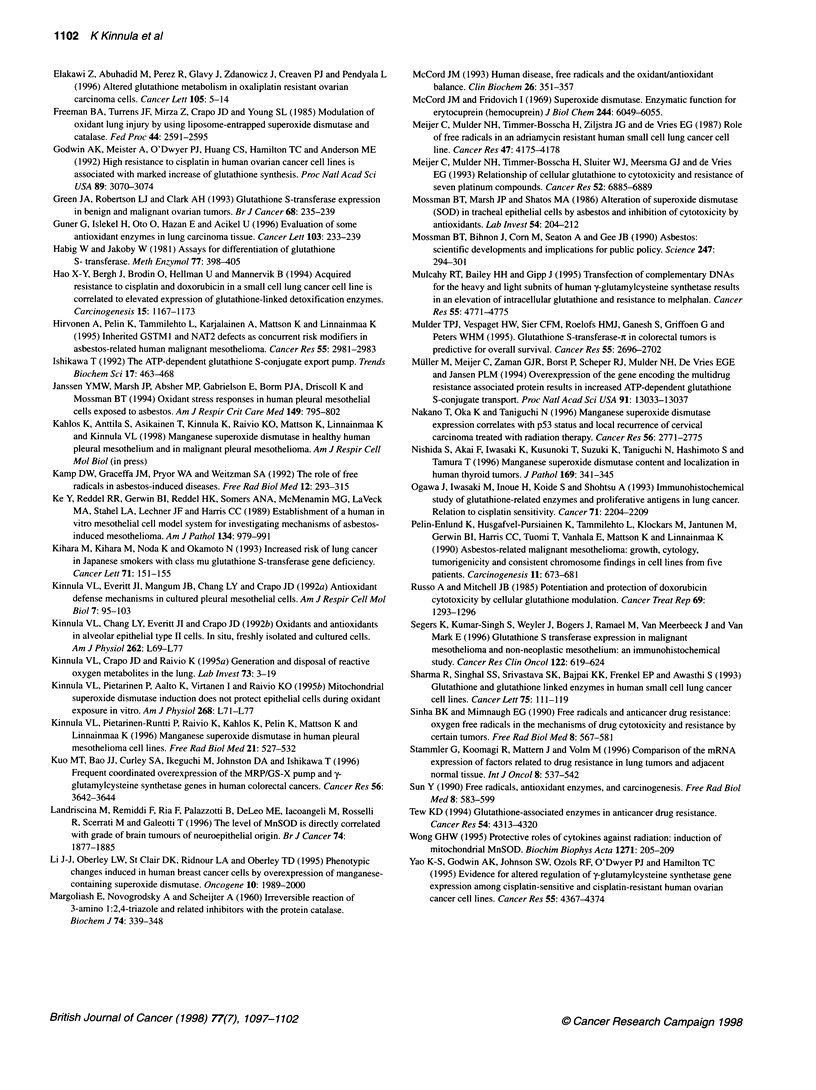# Endogenous antioxidant enzymes and glutathione S-transferase in protection of mesothelioma cells against hydrogen peroxide and epirubicin toxicity.

**DOI:** 10.1038/bjc.1998.182

**Published:** 1998-04

**Authors:** K. Kinnula, K. Linnainmaa, K. O. Raivio, V. L. Kinnula

**Affiliations:** Finnish Institute of Occupational Health, Children's Hospital, University of Helsinki.

## Abstract

**Images:**


British Journal of Cancer (1998) 77(7), 1097–1102
© 1998 Cancer Research Campaign

### Endogenous antioxidant enzymes and glutathione S-transferase in protection of mesothelioma cells against hydrogen peroxide and epirubicin toxicity

K Kinnula[1,2], K Linnainmaa[1], KO Raivio[2] and VL Kinnula[3]

[1]Finnish Institute of Occupational Health, Helsinki; [2]Children's Hospital, University of Helsinki; [3]Department of Internal Medicine, University of Oulu, Finland

**Summary** We have previously shown that cultured malignant mesothelioma cells contain elevated manganese superoxide dismutase (MnSOD) mRNA levels and activities compared with non-malignant mesothelial cells. As many cytotoxic drugs generate both superoxide and hydrogen peroxide, we assessed the relative significance of catalase and the glutathione redox cycle, as well as glutathione S-transferase (GST), in protecting these cells against hydrogen peroxide and epirubicin toxicity. Mesothelioma cell lines containing high (M38K cells) and low (M14K cells) MnSOD, and non-malignant MeT-5A mesothelial cells were selected for the study. M38K cells were the most resistant of these three cell types to hydrogen peroxide (0.1–0.5 mM, 4 h) and epirubicin (0.1–0.5 $\mu$g $ml^{-1}$, 48 h) as judged by lactate dehydrogenase (LDH) release and by high-energy nucleotide (ATP, ADP, AMP) depletion. Total glutathione was higher in M38K cells (63.8 ± 20.3 nnmol $mg^{-1}$ protein) than in M14K (25.2 ± 8.2 nmol $mg^{-1}$) or MeT-5A cells (23.5 ± 4.5 nmol $mg^{-1}$). Furthermore, GST specific activity was higher in M38K cells (111.3 ± 15.8 U $mg^{-1}$) than in M14K cells (77.4 ± 6.6 U $mg^{-1}$) or in MeT-5A cells (68.8 ± 7.6 U $mg^{-1}$). Western blotting indicated the presence of GST-$\pi$ in all these cells, the reactivity again being highest in M38K cells. Depletion of glutathione by buthionine sulphoximine and inhibition of catalase by aminotriazole enhanced hydrogen peroxide toxicity in all cell types, while only the depletion of glutathione increased epirubicin toxicity. We conclude that simultaneous induction of multiple antioxidant enzymes can occur in human mesothelioma cells. In addition to the high MnSOD activity, hydrogen peroxide scavenging antioxidant enzymes, glutathione and GST can partly explain the high hydrogen peroxide and epirubicin resistance of these cells in vitro.

**Keywords:** antioxidant; oxidant; asbestos; superoxide dismutase; glutathione; glutathione S-transferase; mesothelioma

Many chemotherapeutic agents generate reactive oxygen species (ROS), such as superoxide and hydrogen peroxide. However, the role of superoxide dismutases and hydrogen peroxide scavenging antioxidant enzymes (AOEs) in tumour resistance is still unclear. Most previous studies have suggested that levels of the superoxide radical-scavenging superoxide dismutases CuZnSOD and manganese superoxide dismutase (MnSOD) are low in tumours (reviewed by Sun, 1990), and it has been suggested that MnSOD is an anti-cancer gene (Church et al, 1993). Antioxidant and detoxification mechanisms that can be involved in the drug resistance of various tumours include glutathione (Russo and Mitchell 1985; Meijer et al, 1987; Al-Kabban et al, 1990; Sun, 1990; Godwin et al, 1992; Meijer et al, 1993), glutathione S-transferase (GST) (Batist et al, 1986; Green et al, 1993; Hao et al, 1994; Tew, 1994; Ban et al, 1996; Segers et al, 1996), $\gamma$-glutamylcysteine synthetase (Dusre et al, 1989; Bailey et al, 1992; Mulcahy et al, 1995; Yao et al, 1995; Kuo et al, 1996), $\gamma$-glutamyl transpeptidase (Elakawi et al, 1996) and glutathione peroxidase (Meier et al, 1987; Sinha and Mimnaugh, 1990; Ogawa et al, 1993; Hao et al, 1994). Polymorphism of GSTM1 is associated with increased risk for the development of bronchogenic carcinoma (Kihara et al, 1993; Anttila et al, 1994) and mesothelioma (Hirvonen et al, 1995). On the other hand, many tumours and tumour cell lines exhibit high levels of GST-$\pi$, which, at least in certain cases appears to correlate with survival and/or acquired resistance to cytotoxic drugs (Batist et al, 1986; Sharma et al, 1993; Mulder et al, 1995; Cheng, 1997). Only a few studies are available on the significance of catalase in malignant tumours, and most but not all suggest that catalase does not play an important role in tumour resistance (Akman et al, 1989; Sinha and Mimnaugh, 1990).

Human pleural mesothelioma is a resistant and fatal tumour associated with occupational exposure to asbestos fibres, and its pathogenesis is possibly mediated by free radicals (Mossman et al, 1990; Kamp et al, 1992). Asbestos fibres have been shown to cause up-regulation of MnSOD, an important mitochondrial superoxide radical-scavenging enzyme, in bronchial epithelial cells and mesothelial cells (Mossman et al, 1986; Janssen et al, 1994). We have previously shown that human mesothelioma cells contain high MnSOD levels and that these mesothelioma cells are more resistant than non-malignant mesothelial cells against the exogenous oxidant menadione (Kinnula et al, 1996). Because cytotoxic drugs generate both superoxide and hydrogen peroxide, it is likely that hydrogen peroxide-scavenging mechanisms are also involved in the observed resistance of this tumour against oxidant producing chemotherapeutic drugs.

Most previous studies conducted on tumour cells have overlooked the possibility of simultaneous induction of many antioxidant enzymes and have focused only on one, often transfected enzyme. No studies are available on the relative significance of

Received 22 May 1997
Revised 5 September 1997
Accepted 12 September 1997

Correspondence to: VL Kinnula, Department of Internal Medicine, University of Oulu, FIN-90220, Oulu, Finland hydrogen peroxide-scavenging antioxidant enzymes in human mesothelioma. We investigated the relative role of the most important hydrogen peroxide-scavenging AOEs catalase and glutathione peroxidase, glutathione and the detoxification enzyme GST on oxidant and drug resistance in human mesothelioma cells that had been established from the tumours of untreated mesothelioma patients. Cells that had been pretreated with aminotriazole to inactivate catalase or with buthionine sulphoximine to cause glutathione depletion were exposed to either hydrogen peroxide or to epirubicin and were assessed for the development of cell injury. Epirubicin was chosen as it has been shown to generate free radicals intracellularly (Sinha and Mimnaugh, 1990), and it has been widely used in the treatment of mesothelioma.

### METHODS

### Cell culture

Previously characterized (Pelin-Enlund et al, 1990) continuous mesothelioma cell line cells (M38K, M14K) were cultured on uncoated plastic plates in RPMI 1640 medium (Gibco Europe, Paisley, UK) supplemented with heat-inactivated 10% fetal calf serum and the antibiotics streptomycin and penicillin. M38K mesothelioma cell lines contain the highest and M14K cells the lowest MnSOD activities of five mesothelioma cell lines established from the tumours of our untreated mesothelioma patients. Transformed human pleural mesothelial cells (MeT-5A), which are SV-40 virus immortalized, diploid and non-malignant human pleural mesothelial cells (Ke et al, 1989) were a gift from the National Cancer Institute, Bethesda, USA (Dr CC Harris). They were cultured in conditions and medium similar to those for the mesothelioma cell line cells.

### Pretreatments and oxidant exposures

Cells were pretreated with 20 mM aminotriazole (ATZ) for 60 min to inactivate catalase or with 0.2 mM or 1 mM buthionine sulphoximine (BSO) for 16–18 h to inhibit γ-glutamylcysteine synthetase and to cause glutathione depletion. Both these inhibitors are widely used, specific and their effects have been confirmed in earlier investigations (Margoliash et al, 1960; Buckley et al, 1991; Kinnula et al, 1992*ab*). Based on preliminary experiments and cytotoxicity measurements, 0.2 mM BSO concentration was selected for the pretreatment of MeT-5A cells and M14K cells, and 1 mM BSO for the pretreatment of M38K cells. Untreated and pretreated cells after one washing were exposed to 0.1–0.5 mM hydrogen peroxide for 1–4 h or to 0.1–0.5 μg ml–1 epirubicin (0.17–0.85 mM) for 48 h. In the epirubicin experiments, the ATZ treatment was repeated after 24 h for 60 min, and the last 16-h incubation with epirubicin was conducted in the presence of BSO.

### Adenine nucleotides

High energy nucleotide catabolism is an early and sensitive marker of cell injury. To measure the proportion of the high energy nucleotides in the cells, they were preincubated for 16 h with 0.1 mM [$^{14}$C]adenine (specific activity 51–55 mCi mmol$^{-1}$; Amersham International, Amersham, UK). Prelabelled cells were washed and exposed to hydrogen peroxide or epirubicin. After the exposures, the medium was removed and the cells were extracted with 0.4 M perchloric acid. The purine nucleotides (ATP, ADP, AMP) in both the cell extract and the medium and the nucleotide catabolic products (xanthine, hypoxanthine, uric acid) in the medium were separated by thin-layer chromatography as described (Aalto and Raivio, 1990). The results are expressed as per cent distribution of radioactivity (counts per min, c.p.m.) between nucleotides in the cells, nucleotides leaked into the medium and catabolic products in the medium. Normally, the cell membrane of intact cells is not permeable to the cellular high-energy nucleotides, whereas their catabolic products are freely diffusible. Thus, the appearance of nucleotides in the extracellular medium is one way to assess cell membrane injury.

### Lactate dehydrogenase (LDH) release

LDH release into the medium was measured by spectrophotometry using pyruvic acid as the substrate (Bergmeyer, 1974). Total cellular LDH was measured in cell lysates obtained by 1% Triton X-100 treatment.

### Antioxidant enzyme activities and glutathione

Total superoxide dismutase (SOD) activity was measured spectrophotometrically using the method of McCord and Fridovich (1969). MnSOD activity was distinguished from CuZnSOD activity by its resistance to 1 mM potassium cyanide. Glutathione peroxidase activity was analysed by following NADPH oxidation in the presence of t-butylhydroperoxide (Beutler, 1975). Catalase activity was determined using a Clark oxygen electrode as described (Kinnula et al, 1992*b*). Glutathione *S*-transferase (GST) was measured spectrophotometrically using 1 mM 1-chloro-2,4-dinitrobenzene and 1 mM glutathione (Habig and Jakoby, 1981). Enzyme activities are expressed as U mg$^{-1}$ protein. Total glutathione content was determined spectrophotometrically following the reduction of 5,5′-dithiobis (2-nitrobenzoic) acid by NADPH in the presence of glutathione reductase (Beutler, 1975). Glutathione content is expressed as nmol mg$^{-1}$ protein. Proteins were analysed by the micro method of BioRad (Hercules, CA, USA).

### Western blotting

The cells were detached with trypsin, centrifuged and washed with phosphate-buffered saline (PBS). Suspended cells in the sample buffer were boiled for 5 min at 95°C, and 30 μg of protein per lane was applied. The gel was electrophoresed for 1.5 h (90 V), and the protein was transferred onto Hybond enhanced chemiluminescence (ECL) nitrocellulose membrane (Amersham International, Buckinghamshire, UK). Blotted membrane was incubated with rabbit antibody to GST-π (BioPrep, Dublin, Ireland; diluted 1:5000), followed by treatment with donkey anti-rabbit antibody conjugated to horseradish peroxidase (Amersham) (dilution 1:30 000). GST P was detected using the ECL system (Amersham), and luminol excitation was imaged on radiographic film. The amount of the loading was assessed relative to beta actin. Anti-actin antibody (Sigma Chemical, MO, USA) was used in a dilution of 1:20 000.

### Statistical analysis

Data are expressed as mean ± s.d. The groups were compared by analysis of variance and Scheffe's post hoc test; $P < 0.05$ was considered to be significant.

© Cancer Research Campaign 1998

**Figure 1** LDH release from MeT-5A cells, M14K cells and M38K cells after a 4-h exposure to hydrogen peroxide. The cell injury is expressed as relative LDH release compared with non-exposed cells. $^*P < 0.05$ vs non-exposed cells

**Figure 2** Nucleotide depletion, catabolite accumulation and leakage of intact nucleotides of ATZ- and BSO-pretreated and hydrogen peroxide-exposed MeT-5A (**A**), M14K (**B**) and M38K cells (**C**). MeT-5A cells and M14K cells were exposed to 100 μM hydrogen peroxide and M38K cells to 500 μM hydrogen peroxide. Values are means ± s.d. from four to six separate experiments. $^*P < 0.05$ vs hydrogen peroxide-exposed non-treated cells

### RESULTS

M38K cells were most resistant to hydrogen peroxide when assessed by LDH release (Figure 1) or depletion of high-energy nucleotides (not shown). In order to investigate the relative importance of the hydrogen peroxide-scavenging mechanisms catalase and glutathione redox cycle in cell protection, the cells were pretreated with aminotriazole (ATZ) or with buthionine sulphoximide (BSO). After 20 mM ATZ, the activity of catalase decreased by 85% in MeT-5A cells, 82% in M14K cells and 89% in M38K cells (two duplicate experiments). Baseline total glutathione content was 63.8 ± 20.3 nmol $mg^{-1}$ protein ($n$=3) in M38K cells 25.2 ± 8.2 nmol $mg^{-1}$ protein ($n$ = 3) in M14K cells and 23.5 ± 4.5 nmol $mg^{-1}$ protein ($n$ = 3) in MeT-5A cells. BSO at a concentration of 0.2 mM decreased the glutathione concentration in MeT-5A and M14K cells to non-measurable levels. After 0.2 mM BSO treatment, 9.3 ± 7.1 nmol $mg^{-1}$ ($n$ = 3) glutathione was measured in M38K cells (15% of baseline), while the corresponding value after 1 mM BSO treatment was 4.4 ± 0.1 nmol $mg^{-1}$ ($n$ = 3) (7% of baseline). Therefore, 0.2 mM BSO was used for pretreatment of MeT-5A and M14K cells and 1.0 mM BSO for M38K cells. ATZ and 0.2 mM BSO as such did not cause adenine nucleotide depletion in any of these cells, whereas 1 mM BSO induced significant nucleotide catabolism in M14K cells (not shown).

Preliminary experiments indicated that 0.1 mM hydrogen peroxide caused significant nucleotide efflux and catabolism in MeT-5A cells and M14K cells but not in M38K cells; however neither BSO nor ATZ pretreatment could sensitize M38K cells to the effects of 0.1 mM hydrogen peroxide (not shown). Therefore, pretreated MeT-5A cells and M14K cells were exposed to 0.1 mM hydrogen peroxide and pretreated M38K cells to 0.5 mM hydrogen peroxide for 4 h. Hydrogen peroxide enhanced adenine nucleotide catabolism in MeT-5A and M38K cells pretreated with ATZ or BSO, whereas in M14K cells only BSO pretreatment potentiated nucleotide depletion (Figure 2).

In preliminary experiments, epirubicin at a concentration of 0.1 μg $ml^{-1}$ caused significant nucleotide catabolism in MeT-5A cells and M14K cells, but not in untreated or pretreated M38K cells (data not shown). Therefore, pretreated MeT-5A and M14K cells were exposed to 0.1 μg $ml^{-1}$ epirubicin and M38K cells to 0.5 μg $ml^{-1}$ epirubicin for 48 h. BSO potentiated epirubicin toxicity in all three cell types, when assessed by nucleotide catabolism, whereas ATZ pretreatment had no effect on the cellular nucleotide levels in any of these cells (Figure 3).

Intracellular CuZnSOD, MnSOD, glutathione peroxidase and catalase activities in these cells have been (Kinnula et al, 1996) or will be published as part of a study of their gene expression (Kahlos et al, 1998). A summary of those results as well as total glutathione content and total GST activity in the cell lines studied are presented in Table 1. Given that total GST activity was highest in M38K cells and that GST-π isoenzyme is highly involved in the drug resistance of many tumours, GST-π was further assessed by Western blotting. The results indicated that GST-π was most prominently expressed in M38K cells (Figure 4), suggesting that this isoenzyme may play a signficant role in the drug resistance of these cells.

© Cancer Research Campaign 1998

**Figure 3** Nucleotide depletion, catabolite accumulation and leakage of intact nucleotides of ATZ- and BSO-pretreated and epirubicin-exposed (48 h) MeT-5A (**A**), M14K (**B**) and M38K (**C**) cells. The epirubicin concentrations were 0.1 µg ml- for MeT-5A cells and M14K cells,and 0.5 µg ml$^{-1}$ for M38K cells. Values are means±s.d. from four to six separate experiments. $^{*}P <$ 0.05 vs epirubicin-exposed non-treated cells

**Table 1** CuZn superoxide dismutase (CuZnSOD), manganese superoxide dismutase (MnSOD), glutathione peroxidase (Gpx), catalase (Cat) and total glutathione *S*-transferase (GST) activities and intracellular total glutathione content in MeT-5A, M14K and M38K cells

| | MeT-5A | M14K | M38K |
|---|---|---|---|
| CuZnSOD | 11.6 ± 13.7 | 20.1 ± 5.8 | 31.7 ± 11.9 |
| MnSOD | 4.0 ± 3.4 | 10.0 ± 6.0* | 41.0 ± 19.1** |
| Gpx | 1.0 ± 0.4 | 1.9 ± 1.6 | 2.7 ± 2.1 |
| Cat | 13.7 ± 4.9 | 8.1 ± 3.3 | 24.5 ± 7.5** |
| GST | 68.8 ± 7.6 | 77.4 ± 6.6 | 111.3 ± 15.8** |
| Glutathione | 23.5 ± 4.5 | 25.2 ± 8.2 | 63.8 ± 20.3** |

Activities are expressed as U mg$^{-1}$ protein or mU mg$^{-1}$ protein (Gpx). Glutathione concentration is expressed as nmol mg$^{-1}$ protein. Values are means ± s.d., $n$ = 6–8, $^{*}P < 0.05$ vs MeT-5A, $^{**}P < 0.05$ vs MeT-5A and M14K.

**Figure 4** Western blotting of GST-π in MeT-5A mesothelial cells, M14K and M38K mesothelioma cells. The size (kDa) of the molecular marker is indicated on the left in the upper panel. The lower panel shows the densities of GST-π relative to beta actin from two separate experiments conducted in duplicate. The results indicate highest GST-π immunoreactivity in M38K mesothelioma cells

### DISCUSSION

Mesothelioma is highly resistant to all forms of chemotherapy and radiation. We have recently found that human mesothelioma cell lines contain elevated MnSOD mRNA and activity compared with non-malignant mesothelial cells (Kinnula et al, 1996) and that MnSOD is heavily immunostained in human mesothelioma compared with healthy human pleural mesothelium (Kahlos et al, 1998). Out of all mesothelioma cell lines established from the tumours of our untreated patients, M14K cells, which were used here, have the lowest MnSOD activity. Previously, MnSOD has been reported to be low in most tumours and tumour cells (Sinha et al, 1990; Wong, 1995; Guner et al, 1996; Stammler et al, 1996), and it appears to have antiproliferative effects (Church et al, 1993; Li et al, 1995). However, the role of MnSOD in tumours is more complicated than originally assumed. Recent studies have detected high MnSOD levels in colon cancer (Nakano et al, 1996), thyroid cancer (Nishida et al, 1996) and tumours of central nervous origin (Cobbs et al, 1996; Landriscina et al, 1996), and our data on M38K cells show the same result in human mesothelioma (Kinnula et al, 1996). Tumours may have high immunoreactive protein without elevated enzyme activity, but our mesothelioma cells also demonstrated elevated specific activity of MnSOD (Kinnula et al, 1996). As SOD scavenges superoxide radicals to hydrogen peroxide, it may not be the only antioxidant enzyme explaining oxidant or drug resistance of the cells.

One potential mechanism for the high oxidant resistance of mesothelioma cells is simultaneous induction of MnSOD and hydrogen peroxide-scavenging mechanisms, such as catalase and glutathione peroxidase, as well as the availability of glutathione, which is required for the optimal function of the glutathione redox

© Cancer Research Campaign 1998

cycle. M38K cells contained not only the highest activity of MnSOD but also the highest catalase and glutathione levels and somewhat elevated glutathione peroxidase activity. They were also most resistant to hydrogen peroxide and to epirubicin of the cell lines studied. This result is consistent with previous studies, that have shown that a combination of superoxide- and hydrogen peroxide-scavenging AOEs offers better protection against exogenous oxidants than SOD alone (Freeman et al, 1985; Buckley et al, 1987; McCord, 1993; Kinnula et al, 1995*b*). Furthermore, the induction of MnSOD alone is not necessarily protective against oxidant exposure in vitro (Kinnula et al, 1995*a* and *b*), and increased SOD–glutathione peroxidase ratio can increase hydrogen peroxide production and promote cell senescence (De Haan et al, 1996). Therefore, simultaneous induction of SOD and hydrogen peroxide-scavenging pathways may be critical for the resistance of human mesothelioma cells against oxidants and oxidant-producing agents.

Catalase has not usually been connected to drug resistance (Sinha and Mimnaugh, 1990; Coursin et al, 1996). Catalase can be inhibited with ATZ in the presence of hydrogen peroxide but, because complete inhibition is impossible to achieve (Margoliash, 1960; Kinnula et al, 1992*a*), such inhibition studies underestimate the role of catalase in oxidant-exposed cells. In the present study, catalase inhibition potentiated hydrogen peroxide toxicity in the most resistant cell type (M38K), suggesting a function of catalase during heavy oxidant exposure. On the other hand, ATZ had no effect on epirubicin toxicity in any of these cells. This finding does not exclude the importance of catalase in mesothelioma, but it suggests that catalase does not play a central role in the resistance of mesothelioma cells against free radical-generating drugs. In addition, it has to be emphasized that, although ATZ and BSO have been widely used as specific inhibitors of catalase and γ-glutamylcysteine synthetase, the results obtained in the presence of these inhibitors represent indirect data that can theoretically affect other unknown parameters of the cells. Furthermore, the differences between hydrogen peroxide and epirubicin in ATZ- and BSO-treated cells may also be related to the glutathione related drug efflux pump (Ishikawa, 1992; Müller et al, 1994) in these cells.

GST has been widely investigated previously, but its role in various tumours is still incompletely defined. For instance, many but not all studies have shown that tumour cell lines transfected with GST cDNA are resistant to cytotoxic drugs (reviewed by Tew, 1994). In agreement with our study, certain drug-resistant tumour cell lines have been shown to contain elevated levels of multiple antioxidative and detoxification mechanisms, such as total glutathione, glutathione peroxidase and GST (Hao et al, 1994) or γ-glutamylcysteine synthetase (Kuo et al, 1996). In addition, small-cell lung carcinoma cell lines established after chemotherapy have been reported to develop resistance to doxorubicin with a simultaneous increase of glutathione, GST and catalase (De Vries et al, 1989). In our study, M38K cells contained the highest glutathione, total GST activity and GST-π immunoreactivity, suggesting the importance of an ideal balance between glutathione and glutathione-dependent detoxification enzymes in the resistance of these cells to oxidants and at least to some chemotherapeutic compounds. As multiple GST isoenzymes have been characterized, GST-π is not necessarily the only GST type that can be modulated in these cells.

We conclude that antioxidant enzyme levels in mesothelioma cells can be variable. Elevated MnSOD, hydrogen peroxide-scavenging AOEs in combination with glutathione and GST play a more important role than any single antioxidant enzyme in the oxidant and drug resistance of these cells in vitro.

### ACKNOWLEDGEMENTS

The MeT-5A cells were kindly provided by Dr CC Harris (National Cancer Institute, Bethesda, USA). This study has been partly supported by the Finnish Antituberculosis Association Foundation, Cancer Society of Northern Finland and Sigrid Juselius Foundation.

### REFERENCES

Aalto K and Raivio KO (1990) Adenine nucleotide depletion from endothelial cells exposed to xanthine oxidase. *Am J Physiol* **259**: C883–C888

Akman SA, Forrest G, Chu FF and Doroshow JH (1989) Resistance to hydrogen peroxide associated with altered catalase mRNA stability in MCF7 breast cancer cells. *Biochim Biophys Acta* **1009**: 70–74

Al-Kabban M, Stewart MJ, Watson ID and Reglinski J (1990) The effect of doxorubicin on the glutathione content and viability of cultured human lung cancer cell lines A549 and GLC4 210. *Clin Chim Acta* **194**: 121–129

Anttila S, Hirvonen A, Husgafvel-Pursiainen K, Karjalainen A, Nurminen T and Vainio H (1994) Combined effect of CYP1A1 inducibility and *GSTM1* polymorphism on histological type of lung cancer. *Carcinogenesis* **15**: 1133–1135

Bailey HH, Gipp J, Ripple M, Wilding G and Mulcahy T (1992) Increase in γ-glutamylcysteine synthetase activity and steady-state messenger RNA levels in melphalan-resistant DU-145 human prostate cells expressing elevated glutathione levels. *Cancer Res* **52**: 5115–5118

Ban N, Takahashi Y, Takayma T, Kura T, Katahira T, Sakamaki S and Niitsu Y (1996) Transfection of glutathione S-transferase (GST) π-antisense complementary DNA increases the sensitivity of a colon cancer cell line to adriamycin, cisplatin, melphalan, and etoposide. *Cancer Res* **56**: 3577–3582

Batist G, Tulpule A, Sinha BK, Katki AG, Myers CE and Cowan KH (1986) Overexpression of a novel anionic glutathione transferase in multidrug-resistant human breast cancer cells. *J Biol Chem* **261**: 15544–15549

Bergmeyer HU (1974) Lactate dehydrogenase assay with pyruvate and NADH. In *Methods in Enzymology* Vol. 2, Bergmeyer HU (ed.), pp. 574-579. Academic Press, New York

Beutler E (1975) Glutathione peroxidase. In *Red Cell Metabolism: A Manual of Biochemical Methods*, pp. 71–73. Grune & Stratton: New York

Buckley BJ, Tanswell AK and Freeman BA (1987) Liposome-mediated augmentation of catalase in alveolar type II cells protects against $H_2O_2$ injury. *J Appl Physiol* **63**: 359–367

Buckley BJ, Kent RS and Whorton R (1991) Regulation of endothelial cell prostaglandin synthesis by glutathione. *J Biol Chem* **266**: 16659–16666

Cheng X, Kigawa J, Minagawa Y, Kanamori Y, Itamochi H, Okada M and Terakawa N (1997) Glutathione S-Transferase-π expression and glutathione concentration in ovarian carcioma before and after chemotheraby. *Cancer* **79**: 521–527

Church SL, Grant JW, Ridnour LA, Oberley LW, Swanson PE, Meltzer PS and Trent JM (1993) Increased manganese superoxide dismutase expression suppresses the malignant phenotype of human melanoma cells. *Proc Natl Acad Sci USA* **90**: 3113–3117

Cobbs CS, Levi DS, Aldape K and Israel MA (1996) Manganese superoxide dismutase expression in human central nervous system tumors. *Cancer Res* **56**: 3192–3195

Coursin DB, Cihla HP, Oberley TD and Oberley LW (1996) An immunohistochemical analysis of antioxidant and glutathione S-transferase enzyme levels in normal and neoplastic human lung. *Histo Histopathol* **11**: 851–860

De Haan JB, Cristiano F, Iannello R, Bladier C, Kelner MJ and Kola I (1996) Elevation in the ratio of Cu/Zn-superoxide dismutase to glutathione peroxidase activity induces features of cellular senescence and this effect is mediated by hydrogen peroxide. *Hum Mol Gen* **5**: 283–292

De Vries EGE, Meijer C, Timmer-Bosscha H, Berendsen HH, de Leiji L., Scheper RJ and Mulder NH (1989) Resistance mechanism in three human small cell lung cell lines established from one patient during clinical follow up. *Cancer Res* **49**: 4175–4178

Dusre L, Mimnaugh EG, Myers CE and Sinha BK (1989) Potentiation of doxorubicin cytotoxicity by glutathione sulfoximine in multidrug-resistant human breast tumor cells. *Cancer Res* **49**: 511–515

© Cancer Research Campaign 1998

Elakawi Z, Abuhadid M, Perez R, Glavy J, Zdanowicz J, Creaven PJ and Pendyala L (1996) Altered glutathione metabolism in oxaliplatin resistant ovarian carcinoma cells. *Cancer Lett* **105**: 5–14

Freeman BA, Turrens JF, Mirza Z, Crapo JD and Young SL (1985) Modulation of oxidant lung injury by using liposome-entrapped superoxide dismutase and catalase. *Fed Proc* **44**: 2591–2595

Godwin AK, Meister A, O'Dwyer PJ, Huang CS, Hamilton TC and Anderson ME (1992) High resistance to cisplatin in human ovarian cancer cell lines is associated with marked increase of glutathione synthesis. *Proc Natl Acad Sci USA* **89**: 3070–3074

Green JA, Robertson LJ and Clark AH (1993) Glutathione S-transferase expression in benign and malignant ovarian tumors. *Br J Cancer* **68**: 235–239

Guner G, Islekel H, Oto O, Hazan E and Acikel U (1996) Evaluation of some antioxidant enzymes in lung carcinoma tissue. *Cancer Lett* **103**: 233–239

Habig W and Jakoby W (1981) Assays for differentiation of glutathione S- transferase. *Meth Enzymol* **77**: 398–405

Hao X-Y, Bergh J, Brodin O, Hellman U and Mannervik B (1994) Acquired resistance to cisplatin and doxorubicin in a small cell lung cancer cell line is correlated to elevated expression of glutathione-linked detoxification enzymes. *Carcinogenesis* **15**: 1167–1173

Hirvonen A, Pelin K, Tammilehto L, Karjalainen A, Mattson K and Linnainmaa K (1995) Inherited GSTM1 and NAT2 defects as concurrent risk modifiers in asbestos-related human malignant mesothelioma. *Cancer Res* **55**: 2981–2983

Ishikawa T (1992) The ATP-dependent glutathione S-conjugate export pump. *Trends Biochem Sci* **17**: 463–468

Janssen YMW, Marsh JP, Absher MP, Gabrielson E, Borm PJA, Driscoll K and Mossman BT (1994) Oxidant stress responses in human pleural mesothelial cells exposed to asbestos. *Am J Respir Crit Care Med* **149**: 795–802

Kahlos K, Anttila S, Asikainen T, Kinnula K, Raivio KO, Mattson K, Linnainmaa K and Kinnula VL (1998) Manganese superoxide dismutase in healthy human pleural mesothelium and in malignant pleural mesothelioma. *Am J Respir Cell Mol Biol* (in press)

Kamp DW, Graceffa JM, Pryor WA and Weitzman SA (1992) The role of free radicals in asbestos-induced diseases. *Free Rad Biol Med* **12**: 293–315

Ke Y, Reddel RR, Gerwin BI, Reddel HK, Somers ANA, McMenamin MG, LaVeck MA, Stahel LA, Lechner JF and Harris CC (1989) Establishment of a human in vitro mesothelial cell model system for investigating mechanisms of asbestos-induced mesothelioma. *Am J Pathol* **134**: 979–991

Kihara M, Kihara M, Noda K and Okamoto N (1993) Increased risk of lung cancer in Japanese smokers with class mu glutathione S-transferase gene deficiency. *Cancer Lett* **71**: 151–155

Kinnula VL, Everitt JI, Mangum JB, Chang LY and Crapo JD (1992*a*) Antioxidant defense mechanisms in cultured pleural mesothelial cells. *Am J Respir Cell Mol Biol* **7**: 95–103

Kinnula VL, Chang LY, Everitt JI and Crapo JD (1992*b*) Oxidants and antioxidants in alveolar epithelial type II cells. In situ, freshly isolated and cultured cells. *Am J Physiol* **262**: L69–L77

Kinnula VL, Crapo JD and Raivio K (1995*a*) Generation and disposal of reactive oxygen metabolites in the lung. *Lab Invest* **73**: 3–19

Kinnula VL, Pietarinen P, Aalto K, Virtanen I and Raivio KO (1995*b*) Mitochondrial superoxide dismutase induction does not protect epithelial cells during oxidant exposure in vitro. *Am J Physiol* **268**: L71–L77

Kinnula VL, Pietarinen-Runtti P, Raivio K, Kahlos K, Pelin K, Mattson K and Linnainmaa K (1996) Manganese superoxide dismutase in human pleural mesothelioma cell lines. *Free Rad Biol Med* **21**: 527–532

Kuo MT, Bao JJ, Curley SA, Ikeguchi M, Johnston DA and Ishikawa T (1996) Frequent coordinated overexpression of the MRP/GS-X pump and γ-glutamylcysteine synthetase genes in human colorectal cancers. *Cancer Res* **56**: 3642–3644

Landriscina M, Remiddi F, Ria F, Palazzotti B, DeLeo ME, Iacoangeli M, Rosselli R, Scerrati M and Galeotti T (1996) The level of MnSOD is directly correlated with grade of brain tumours of neuroepithelial origin. *Br J Cancer* **74**: 1877–1885

Li J-J, Oberley LW, St Clair DK, Ridnour LA and Oberley TD (1995) Phenotypic changes induced in human breast cancer cells by overexpression of manganese-containing superoxide dismutase. *Oncogene* **10**: 1989–2000

Margoliash E, Novogrodsky A and Scheijter A (1960) Irreversible reaction of 3-amino 1:2,4-triazole and related inhibitors with the protein catalase. *Biochem J* **74**: 339–348

McCord JM (1993) Human disease, free radicals and the oxidant/antioxidant balance. *Clin Biochem* **26**: 351–357

McCord JM and Fridovich I (1969) Superoxide dismutase. Enzymatic function for erytocuprein (hemocuprein) *J Biol Chem* **244**: 6049–6055.

Meijer C, Mulder NH, Timmer-Bosscha H, Ziljstra JG and de Vries EG (1987) Role of free radicals in an adriamycin resistant human small cell lung cancer cell line. *Cancer Res* **47**: 4175–4178

Meijer C, Mulder NH, Timmer-Bosscha H, Sluiter WJ, Meersma GJ and de Vries EG (1993) Relationship of cellular glutathione to cytotoxicity and resistance of seven platinum compounds. *Cancer Res* **52**: 6885–6889

Mossman BT, Marsh JP and Shatos MA (1986) Alteration of superoxide dismutase (SOD) in tracheal epithelial cells by asbestos and inhibition of cytotoxicity by antioxidants. *Lab Invest* **54**: 204–212

Mossman BT, Bihnon J, Corn M, Seaton A and Gee JB (1990) Asbestos: scientific developments and implications for public policy. *Science* **247**: 294–301

Mulcahy RT, Bailey HH and Gipp J (1995) Transfection of complementary DNAs for the heavy and light subnits of human γ-glutamylcysteine synthetase results in an elevation of intracellular glutathione and resistance to melphalan. *Cancer Res* **55**: 4771–4775

Mulder TPJ, Vespaget HW, Sier CFM, Roelofs HMJ, Ganesh S, Griffoen G and Peters WHM (1995). Glutathione S-transferase-π in colorectal tumors is predictive for overall survival. *Cancer Res* **55**: 2696–2702

Müller M, Meijer C, Zaman GJR, Borst P, Scheper RJ, Mulder NH, De Vries EGE and Jansen PLM (1994) Overexpression of the gene encoding the multidrug resistance associated protein results in increased ATP-dependent glutathione S-conjugate transport. *Proc Natl Acad Sci USA* **91**: 13033–13037

Nakano T, Oka K and Taniguchi N (1996) Manganese superoxide dismutase expression correlates with p53 status and local recurrence of cervical carcinoma treated with radiation therapy. *Cancer Res* **56**: 2771–2775

Nishida S, Akai F, Iwasaki K, Kusunoki T, Suzuki K, Taniguchi N, Hashimoto S and Tamura T (1996) Manganese superoxide dismutase content and localization in human thyroid tumors. *J Pathol* **169**: 341–345

Ogawa J, Iwasaki M, Inoue H, Koide S and Shohtsu A (1993) Immunohistochemical study of glutathione-related enzymes and proliferative antigens in lung cancer. Relation to cisplatin sensitivity. *Cancer* **71**: 2204–2209

Pelin-Enlund K, Husgafvel-Pursiainen K, Tammilehto L, Klockars M, Jantunen M, Gerwin BI, Harris CC, Tuomi T, Vanhala E, Mattson K and Linnainmaa K (1990) Asbestos-related malignant mesothelioma: growth, cytology, tumorigenicity and consistent chromosome findings in cell lines from five patients. *Carcinogenesis* **11**: 673–681

Russo A and Mitchell JB (1985) Potentiation and protection of doxorubicin cytotoxicity by cellular glutathione modulation. *Cancer Treat Rep* **69**: 1293–1296

Segers K, Kumar-Singh S, Weyler J, Bogers J, Ramael M, Van Meerbeeck J and Van Mark E (1996) Glutathione S transferase expression in malignant mesothelioma and non-neoplastic mesothelium: an immunohistochemical study. *Cancer Res Clin Oncol* **122**: 619–624

Sharma R, Singhal SS, Srivastava SK, Bajpai KK, Frenkel EP and Awasthi S (1993) Glutathione and glutathione linked enzymes in human small cell lung cancer cell lines. *Cancer Lett* **75**: 111–119

Sinha BK and Mimnaugh EG (1990) Free radicals and anticancer drug resistance: oxygen free radicals in the mechanisms of drug cytotoxicity and resistance by certain tumors. *Free Rad Biol Med* **8**: 567–581

Stammler G, Koomagi R, Mattern J and Volm M (1996) Comparison of the mRNA expression of factors related to drug resistance in lung tumors and adjacent normal tissue. *Int J Oncol* **8**: 537–542

Sun Y (1990) Free radicals, antioxidant enzymes, and carcinogenesis. *Free Rad Biol Med* **8**: 583–599

Tew KD (1994) Glutathione-associated enzymes in anticancer drug resistance. *Cancer Res* **54**: 4313–4320

Wong GHW (1995) Protective roles of cytokines against radiation: induction of mitochondrial MnSOD. *Biochim Biophys Acta* **1271**: 205–209

Yao K-S, Godwin AK, Johnson SW, Ozols RF, O'Dwyer PJ and Hamilton TC (1995) Evidence for altered regulation of γ-glutamylcysteine synthetase gene expression among cisplatin-sensitive and cisplatin-resistant human ovarian cancer cell lines. *Cancer Res* **55**: 4367–4374

© Cancer Research Campaign 1998